# Serum MiR-4687-3p Has Potential for Diagnosis and Carcinogenesis in Non-small Cell Lung Cancer

**DOI:** 10.3389/fgene.2020.597508

**Published:** 2020-11-23

**Authors:** Man Liu, Qiufang Si, Songyun Ouyang, Zhigang Zhou, Meng Wang, Chunling Zhao, Ting Yang, Yulin Wang, Xue Zhang, Wenbo Xie, Liping Dai, Jitian Li

**Affiliations:** ^1^Henan Institute of Medical and Pharmaceutical Sciences, Academy of Medical Sciences, Zhengzhou University, Zhengzhou, China; ^2^Laboratory of Molecular Biology, Henan Luoyang Orthopedic Hospital (Henan Provincial Orthopedic Hospital), Zhengzhou, China; ^3^BGI College, Zhengzhou University, Zhengzhou, China; ^4^Department of Respiratory and Sleep Medicine, The First Affiliated Hospital in Zhengzhou University, Zhengzhou, China; ^5^Department of Radiology, The First Affiliated Hospital in Zhengzhou University, Zhengzhou, China; ^6^Henan Key Laboratory of Tumor Epidemiology & State Key Laboratory of Esophageal Cancer Prevention, Zhengzhou University, Zhengzhou, China; ^7^Department of Computer Science, College of Engineering, University of Texas at El Paso, El Paso, TX, United States

**Keywords:** NSCLC, miR-4687-3p, microarray, bioinformatics, biomarker

## Abstract

The lack of a useful biomarker partly contributes to the increased mortality of non-small cell lung cancer (NSCLC). MiRNAs have become increasingly appreciated in diagnosis of NSCLC. In the present study, we used microarray to screen 2,549 miRNAs in serum samples from the training cohort (NSCLC, *n* = 10; the healthy, *n* = 10) to discover differentially expressed miRNAs (DEMs). Quantitative reverse-transcription polymerase chain reaction (qRT-PCR) assay was applied to validate the expression level of selected overexpressed DEMs of NSCLC in a validation cohort (NSCLC, *n* = 30; the healthy, *n* = 30). Area under the receiver operating characteristic curve (AUC) was performed to evaluate diagnostic capability of the DEMs. The expression of the miRNAs in tissues was analyzed based on the TCGA database. Subsequently, the target genes of the miR-4687-3p were predicted by TargetScan. Gene Ontology (GO), and Kyoto Encyclopedia of Genes and Genomes (KEGG) pathway enrichment analysis were tested by R software (ClusterProfiler package). NSCLC cells were transfected with inhibitor or mimic to down-regulate or up-regulate the miR-4687-3p level. The function of miR-4687-3p on proliferation, invasion, and migration of lung cancer cells were investigated through CCK-8 and Transwell assays, respectively. In the results, we identified serum miR-4687-3p that provided a high diagnostic accuracy of NSCLC (AUC = 0.679, 95%CI: 0.543–0.815) in the validation cohort. According to the TCGA database, we found that the miR-4687-3p level was significantly higher in NSCLC tissues than in normal lung tissues (*p* < 0.05). GO and KEGG pathway enrichment analysis showed that postsynaptic specialization and TGF-β signaling pathway were significantly enriched. Down-regulation of miR-4687-3p could suppress the proliferation, invasion, and migration of the NSCLC cells, compared with inhibitor negative control (NC). Meanwhile, overexpression of miR-4687-3p could promote the proliferation, invasion, and migration of the NSCLC cells compared with mimic NC. As a conclusion, our study first discovered that serum miR-4687-3p might have clinical potential as a non-invasive diagnostic biomarker for NSCLC and play an important role in the development of NSCLC.

## Introduction

Lung cancer is the most common malignancy and the leading cause of malignancy-related death in the world, with an estimated 2.3 million new cases and 1.4 million deaths (Siegel and Miller, 2019). In addition, lung cancer occupied the highest incidence and mortality in numerous malignant cancers in China (Zheng et al., 2019). In the absence of significant symptoms and practical diagnostic methods, most patients are at advanced disease (Zheng et al., 2019). In terms of the pathological type, non-small cell lung cancer (NSCLC) accounts for about 85% of lung cancer. Therefore, exploring early detection methods for NSCLC is crucial to reduce NSCLC-related deaths. Low-dose spiral computed tomography (LDCT) can detect early lung cancer patients and reduce lung cancer-related mortality effectively, and pathological diagnosis provides the gold standard for the diagnosis of lung cancer. However, high false positive rate limits the application of LDCT in clinic. Moreover, the pathological diagnosis belongs to the invasive detection method, which causes great pain to the patients. Hence, searching for a convenient and non-invasive method for the diagnosis of NSCLC can play an essential role in improving the prognosis of NSCLC (Grilley-Olson et al., 2013). Serum contains types of molecular markers, and serum test is non-invasive and cheap, so it is one of the best sources of biomarkers. Moreover, many serum markers have been applied to detect lung cancer in clinic, such as CEA and SCC. However, the serum biomarkers are always limited by low sensitivity and specificity.

MiRNA, a small non-protein coding RNA, targets to 3’ UTR of mRNA, degrades the mRNA and alters the expression of the gene and protein encoded (Bartel, 2004). MiRNA could be released by cancer cells into serum, plasma, or other body fluids to a participant in carcinogenesis, which have been reported in multiple cancers, such as hepatoma carcinoma, NSCLC (Zhang et al., 2015; Zhao et al., 2016; Usuba et al., 2019; Valihrach et al., 2019). As a biomarker, serum miRNAs have particular merits: resistant to RNase digestion and repeated freezing and thawing (Chen et al., 2008; Mitchell et al., 2008).

Several serum miRNAs with differential expression in patients with NSCLC and the healthy were reported recently, such as miR-16 (Fan et al., 2016), miR-504 (Szpechcinski et al., 2019), and miR-21 (Zhao et al., 2015). As a favorable biomarker for the disease, the miRNA should possess certain biological functions. However, most previous studies merely expound the differential expression of the miRNA in NSCLC and the healthy, but rarely explore the biological function of the miRNA in NSCLC.

**FIGURE 1:**
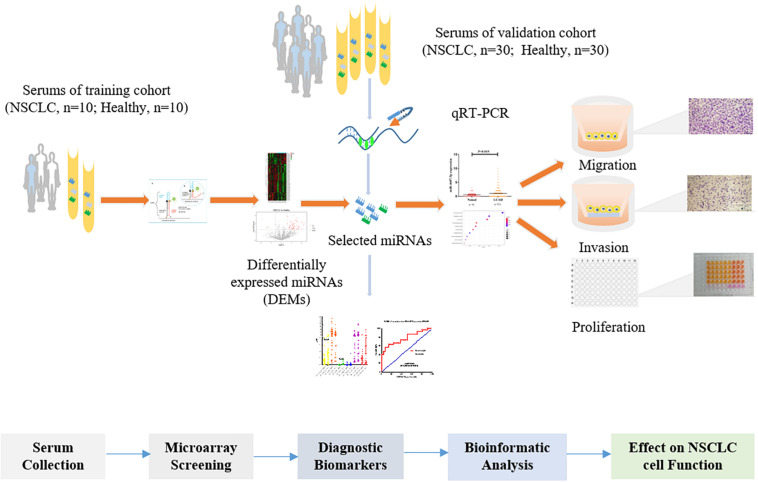
An overview of the workflow of the study design.

The present study screened serum miRNA expression profiles (2,549 miRNAs) in 20 serum samples, and the selected miRNAs were validated in a cohort (NSCLC, *n* = 30; the healthy, *n* = 30). Moreover, we identified the expression level of tissue miR-4687-3p in lung adenocarcinoma (LUAD) and squamous cell lung carcinoma (LUSC) based on the TCGA database. Furthermore, we explored its function on the proliferation, invasion, and migration of NSCLC cells. The study design is illustrated in Figure 1.

## Materials and Methods

### Study Participants

In this study, 50 patients with NSCLC (Table 1) were recruited at the time of diagnosis, before any medical or surgical treatment, from the First Affiliated Hospital of Zhengzhou University (Zhengzhou, China) between January 2018 and December 2018. Moreover, 50 healthy participants (Table 1) were collected from health examination populations without pulmonary-related diseases or other cancers from Zhengzhou Hospital of Traditional Chinese Medicine (Zhengzhou, China). Written informed consent was obtained from all subjects (NSCLC patients and healthy controls) before the study began, and the study protocol was approved by Medical Ethics Committee of Zhengzhou University (Zhengzhou, China). The detailed information of the participants from training (NSCLC, *n* = 20; the healthy, *n* = 20) and validation cohort (NSCLC, *n* = 30; the healthy, *n* = 30) were illustrated in Supplementary Tables 1, 2 respectively.

**TABLE 1:** Characteristics of study participants in the training and validation cohort.

**Factors**	**Subgroup**	**Training cohort No. of participants (%)**	**Validation cohort No. of participants (%)**
**Healthy**		20	30
Age, year			
	Mean	53.9	54.1
		4.3	12.0
Sex			
	Male	10 (50%)	14 (46.7%)
	Female	10 (50%)	16 (53.3%)
**NSCLC**		20	30
Age, year			
	Mean	56.1	60.8
		5.9	9.5
Sex			
	Male	10 (50%)	19 (63.3%)
	Female	10 (50%)	11 (36.7%)
Histopathological type			
	LUSC	6 (30%)	13 (43.3%)
	LUAD	14 (70%)	17 (56.7%)
Stage			
	I+II	5 (25%)	1 (3%)
	III+IV	10 (50%)	24 (80%)
	Undetermined	5 (25%)	5 (17%)

**LUSC, Lung Squamous Carcinoma; LUAD, Lung Adenocarcinoma.**

**TABLE 2:** Six differentially overexpressed serum miRNAs in NSCLC.

**Name**	***P*-value**	**Fold change**	**NSCLC (Raw)**	**Healthy (Raw)**
MiR-1915-5p	0.027	3.3	10.2	6.6
MiR-432-3p	0.042	2.3	25.5	18.6
MiR-4488	0.022	2.5	23.7	16.4
MiR-4687-3p	0.010	1.5	470.8	301.1
MiR-520a-5p	0.040	3.1	28.3	23.2
MiR-6087	0.001	1.5	2764.2	1772.4

After centrifugation at 3,000 rpm for 5 min, the serum sample was divided into 500 μL/tube and stored at −80°C immediately to avoid repeated freezing and thawing.

### Microarray Assay

Human miRNA microarray 2.0 from Agilent Technologies (Santa Clara, CA) including 2,549 miRNAs were applied to screen candidate miRNAs for diagnosing NSCLC in 20 serum samples. The aim was to reduce bias caused by sample heterogeneity, and sera from two participants were merged as a serum sample. Total RNA was harvested using TRIzol (Invitrogen) and the RNeasy Mini Kit (Qiagen), and then labeled and hybridized on the human miRNA microarray. After microarray washing and scanning, the data were extracted with Agilent Feature Extraction Software. The assay was conducted by KangChen Bio-tech (Shanghai, China).

### RNA Extraction and qRT-PCR

Total RNA was harvested from the serum samples using TRIzol (Takara, Japan) and then used to synthesize cDNA by the stem-loop method using the PrimeScript RT Master Mix Kit (Promega, United States). qRT-PCR was carried out by using an ABI Q3 system (Applied Biosystems, Foster City, CA). The primers used in the study were purchased from Sangya Corporation (Sangya, China) or RiboBio Corporation (Ribo, China). Thereinto, U6 primer was using the Bulge-Loop miRNA qRT-PCR Primer Set (RiboBio, China) according to the manufacturer’s instructions.

U6 and serum miR-484 (Zhou et al., 2015) expression were used as a stable endogenous control for normalization in cells and serum samples, respectively.

### TCGA Data Analysis

The Cancer Genome Atlas (TCGA) contains multiple primary cancer and corresponding normal samples, including lung cancer. There are two datasets for lung cancer (TCGA LUAD and TCGA LUSC), which include LUAD (*n* = 521) with corresponding normal tissues (*n* = 46), and LUSC (*n* = 478) with corresponding normal tissues (*n* = 45), respectively. After we downloaded and normalized the non-transcriptome expression data of TCGA lung cancer, we compared tissue miR-4687-3p expression in LUAD, LUSC, and corresponding normal tissue^1^.

### Target Prediction and Enrichment Analysis

TargetScan was used to perform the target prediction of miR-4687-3p. For the target genes, GO and KEGG enrichment analysis were conducted by R software (ClusterProfiler package) to clarify the potential function of miRNA.

### Cell Culture

Human NSCLC cell lines A549, PC-9, H1299, H1975 were cultured in RPMI 1640 (BI, Israel) medium with 10% FBS (BI, Israel) and CALU-3 in DMEM (BI, Israel) medium with 10% FBS. These cell lines were grown in a cell incubator, and the medium was replaced every 2 days.

### MiRNA Transfection

MiR-4687-3p mimics, inhibitors, and corresponding controls were obtained from RiboBio (RiboBio, China). Cells were added into six-well plates with 2.5 × 10^5^ cells per well and cultured overnight. When the cell confluence reached 30–40%, cells were transfected with inhibitor negative control (concentration: 100 nM), miR-4687-3p inhibitor (concentration: 100 nM), or mimic negative control (concentration: 50 nM), miR-4687-3p mimic (concentration: 50 nM), respectively. Lipofectamine^TM^3000 (Invitrogen, United States) was used as the transfection reagent.

### CCK-8 Cell Proliferation Assay

Cells were seeded into 96-well plates (3–4 × 10^3^ cells/well) and supplemented with corresponding transfection reagents after 6–8 h. Each group included six parallel wells. After cells were incubated at 37°C for 24, 48, and 72 h, 100 μL CCK-8 mixture (Dalian Mellon, China) (CCK-8: DMEM/RPMI1640 medium = 1:9) was added into the wells. The absorbance at 450 nm was measured by microplate reader.

### Transwell Invasion and Migration Assay

After incubation for 24 h, the transfected cells were trypsinized and resuspended in DMEM/RPMI 1640 without FBS. Then 70 μL of Matrigel (BD, United States) (Matrigel: DMEM/RPMI1640 medium = 1:9) was added into the upper chamber and the transwell plate (Corning, United States) was placed into the incubator for 30 min. After that, excess unset Matrigel was removed followed by adding 200 μL cell suspension (5 × 10^4^ CALU-3 cells or 10^5^ PC-9 cells) into the upper chamber when the lower chamber was filled with DMEM/RPMI1640 medium containing 20% FBS. The transwell chamber was then placed into a 24-well transwell plate. After 24 h of incubation, the chamber was washed by PBS, fixed with 4% paraformaldehyde for 15 min, and stained with 0.1% crystal violet (Solarbio, China) for 30 min. Finally, we photographed the cells under the microscope and counted the number of cells that passed through the filter membrane in five random fields.

The process of migration assay was similar to the invasion assay, except for laying Matrigel into the chamber. In addition, in the migration assay, we added 1.5 × 10^4^ CALU-3 or 5 × 10^4^ PC-9 cells into the upper chamber, respectively.

### Statistical Analysis

The differential expression of miRNAs between NSCLC and healthy group in the microarray analysis and qRT-PCR analysis was analyzed by Mann-Whitney unpaired test. Independent sample T test and analysis of variance (ANOVA) were used to analyze the data from cell function experiments. GraphPad prism 5 software (La Jolla, CA, United States) was used to present the data. The predicted probability of being diagnosed with NSCLC was used as a surrogate marker to construct receiver operating characteristic (ROC) curve. Area under the ROC curve (AUC) was used as an accuracy index for evaluating the diagnostic performance of the selected miRNAs. The ROC and regression analysis were performed by SPSS 19.0 software (IBM, United States). Two sides *P* < 0.05 was considered statistically significant.

## Results

### MiRNA Screening

**FIGURE 2:**
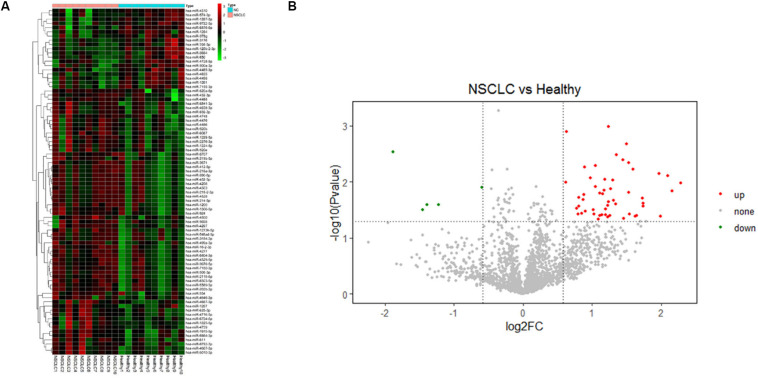
**(A)** Hierarchical clustering and **(B)** Volcano Plot of the differentially expressed miRNAs (DEMs) in the comparison of NSCLC and the healthy (fold change > 1.5, *p* < 0.05).

A microarray, including probes for 2,549 human miRNAs, was used to screen the significant differential miRNAs (DEMs) (fold change > 1.5, *p* < 0.05) between the NSCLC and the healthy, which were displayed through the hierarchical clustering and Volcano Plot in Figure 2. We obtained 53 up-regulated DEMs and five down-regulated DEMs in NSCLC, compared with the healthy (Supplementary Table 3). Taken fold change > 1.5, *p* < 0.05, raw value > 5 as a criterion, we screened six miRNAs (Tables 2, 3) significantly up-regulated in NSCLC, compared with the healthy. Then, we detected the expression of the selected miRNAs in the validation cohort via qRT-PCR.

**TABLE 3:** Primer sequence of the selected six miRNAs and reference miRNA.

**Name**	**Specific reverse primer**	**Forward primer**	**Reverse primer**
MiR-1915-5p	GTCGTATCCAGTGCAGGGTCCGAGGTATTCGCACTGGATACGACGGCCCG	ATATCGACCTTGCCTTGCTGCC	AGTGCAGGGTCCGAGGTATT
MiR-432-3p	GTCGTATCCAGTGCAGGGTCCGAGGTATTCGCACTGGATACGACAGACAT	AATCCGCTGGATGGCTCCTCC	AGTGCAGGGTCCGAGGTATT
MiR-4488	GTCGTATCCAGTGCAGGGTCCGAGGTATTCGCACTGGATACGACCGCCGG	ATATATCGAGGGGGCGGGCT	AGTGCAGGGTCCGAGGTATT
MiR-4687-3p	GTCGTATCCAGTGCAGGGTCCGAGGTATTCGCACTGGATACGACGCCTGC	ATATCCGTGGCTGTTGGAGGGG	AGTGCAGGGTCCGAGGTATT
MiR-520a-5p	GTCGTATCCAGTGCAGGGTCCGAGGTATTCGCACTGGATACGACAGAAAG	CCGCGCTCCAGAGGGAAGTA	AGTGCAGGGTCCGAGGTATT
MiR-6087	GTCGTATCCAGTGCAGGGTCCGAGGTATTCGCACTGGATACGACGCTCGC	ATATATCGTGAGGCGGGGGG	AGTGCAGGGTCCGAGGTATT
MiR-484	CTCAACTGGTGTCGTGGAGTCGGCAATTCAGTTGAGATCGGGAG	ACACTCCAGCTGGGTCAGGCTC AGTCCCCT	TGGTGTCGTGGAGTCG

### Expression of the Six Selected MiRNAs

**FIGURE 3:**
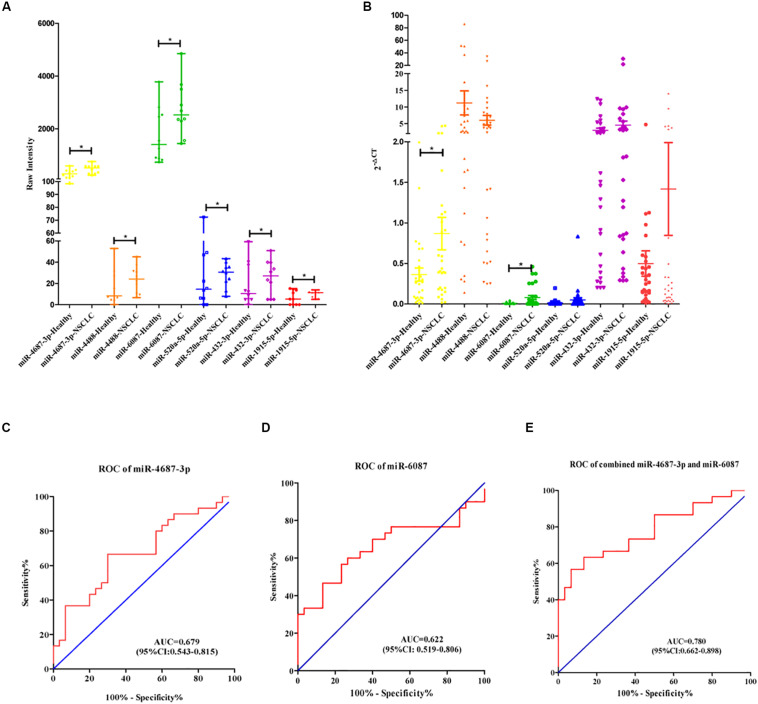
**(A)** Scatter diagram exhibited the row intensity of six miRNAs in NSCLC serum samples (*n* = 10) and healthy serum samples (*n* = 10) in training cohort. **(B)** Scatter diagram exhibited the relative expression of six miRNAs of NSCLC serum samples (*n* = 30) and the healthy serum samples (*n* = 30) in validation cohort. **(C–E)** Receiver operating characteristic curve analysis of miR-4687-3p, miR-6087, and combined with the two miRNAs for NSCLC diagnosis in the validation cohort.

Expression of the selected six individual miRNAs according to the training cohort (Figure 3A) was evaluated with qRT-PCR in 60 serum samples (NSCLC, *n* = 30; the healthy, *n* = 30) (Figure 3B). Serum miR-4687-3p and miR-6087 owned a high expression level in NSCLC compared with the healthy. In addition, miR-4687-3p provided a higher diagnostic accuracy of NSCLC than the other five miRNAs (AUC = 0.679, 95% CI: 0.543–0.815) (Figures 3C,D). The AUC of the combination of miR-4687-3p and miR-6087 using logistic regression model reached 0.780 (95%CI: 0.662–0.898) (Figure 3E).

### The Expression of Tissue MiR-4687-3p Was Validated in TCGA Database

**FIGURE 4:**
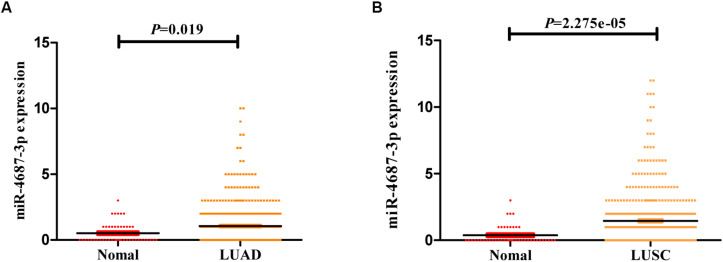
**(A)** The expression of miR-4687-3p in LUAD tissues (*n* = 521) and normal lung tissues (*n* = 46). **(B)** The expression of miR-4687-3p in LUSC tissues (*n* = 478) and normal lung tissues (*n* = 45). Data were from TCGA database. LUAD, Lung Adenocarcinoma; LUSC, Lung Squamous Cell Carcinomas.

According to the TCGA database of NSCLC, the expression of the tissue miR-4687-3p was higher both in LUAD (Figure 4A) and LUSC (Figure 4B) than corresponding normal tissues (*p* < 0.05).

### GO and KEGG Pathway Enrichment

**FIGURE 5:**
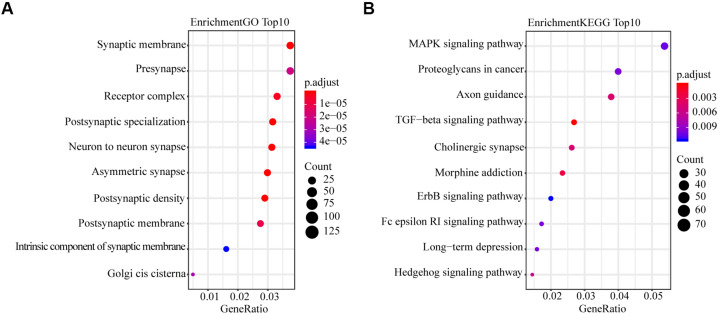
**(A)** Plot of the enriched GO terms. Go enrichment analysis for miR-4687-3p-related mRNAs. **(B)** Plot of the KEGG pathways. KEGG pathway enrichment analysis for miR-4687-3p-related mRNAs. The bubble color and size represent enrichment significance and the number of target mRNAs enriched in a GO term or pathway, respectively. *p* < 0.05 was used as the threshold to select GO and KEGG terms.

To explore the mechanism in the process of miR-4687-3p regulating NSCLC cell progression, we predicted the target genes of miR-4687-3p by using TargetScan, which provided 3,851 target genes. Meanwhile, we proceeded with GO and KEGG pathway enrichment analysis for the 3,851 target genes, which showed that postsynaptic specialization and TGF-β signaling pathway were significantly enriched (Figures 5A,B).

### MiR-4687-3p Promoted the Proliferation, Invasion, and Migration of the NSCLC Cells

**FIGURE 6:**
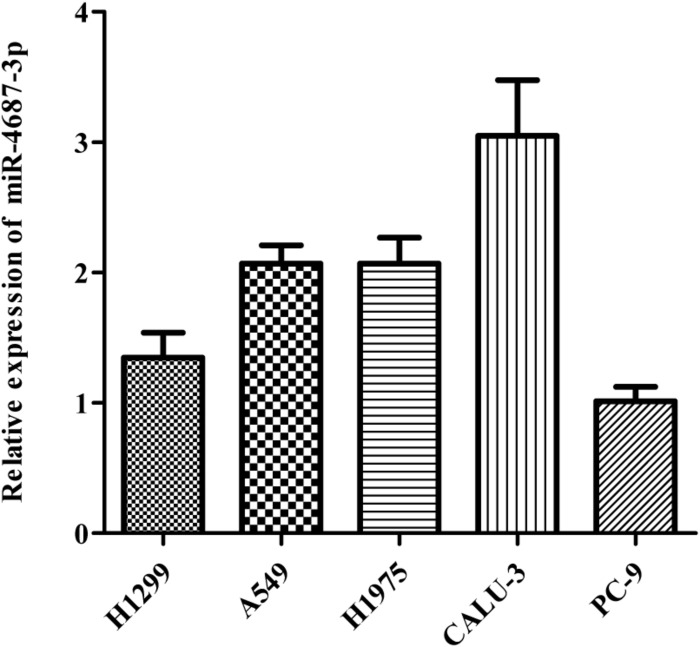
Comparison of miR-4687-3p in five NSCLC cells. The PC-9 cells were used as the control.

MiR-4687-3p expression was detected in five NSCLC cell lines (A549, PC-9, CALU-3, H1299, H1975), which showed that the CALU-3 and PC-9 cells were the highest and lowest observed expression of miR-4687-3p, respectively (Figure 6). Therefore, CALU-3 and PC-9 cells were selected for the next experiments.

**FIGURE 7:**
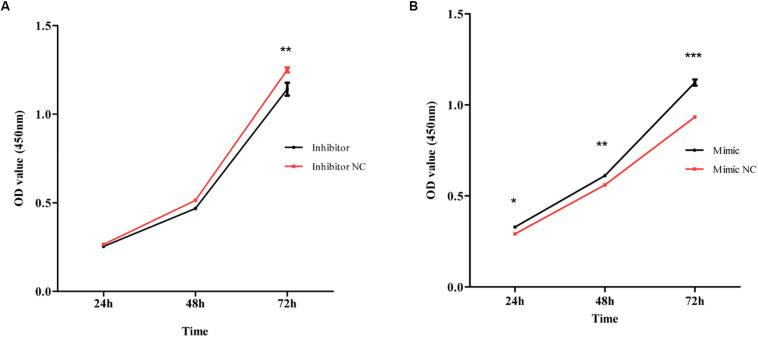
**(A)** CALU-3 cells were transfected with inhibitor control and miR-4687-3p inhibitor, respectively. Cell proliferation was evaluated by the CCK-8 assay at 24, 48, and 72 h. **(B)** PC-9 Cells were transfected with mimic control and miR-4687-3p mimic, respectively. Cell proliferation was evaluated by the CCK-8 assay at 24, 48, and 72 h. *p < 0.05, **p < 0.01, ***p < 0.001.

**FIGURE 8:**
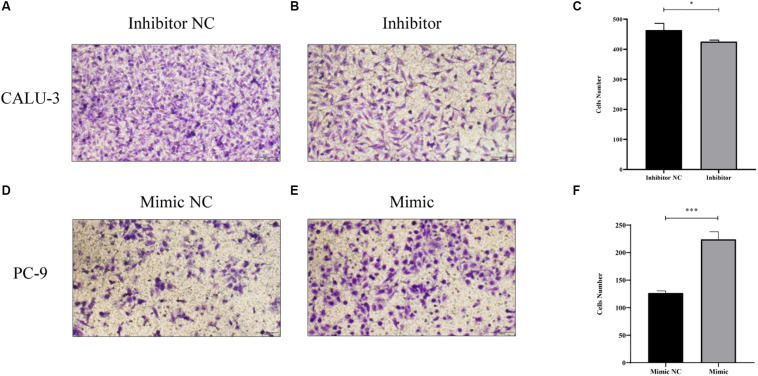
**(A–C)** Transwell invasion assay with Matrigel was performed in miR-4687-3p inhibitor or inhibitor control transfected CALU-3 cells (Magnification ×400). **(D–F)** Transwell invasion assay with Matrigel was performed in miR-4687-3p mimic or mimic control transfected PC-9 cells (Magnification ×400). *p < 0.05, ***p < 0.001.

**FIGURE 9:**
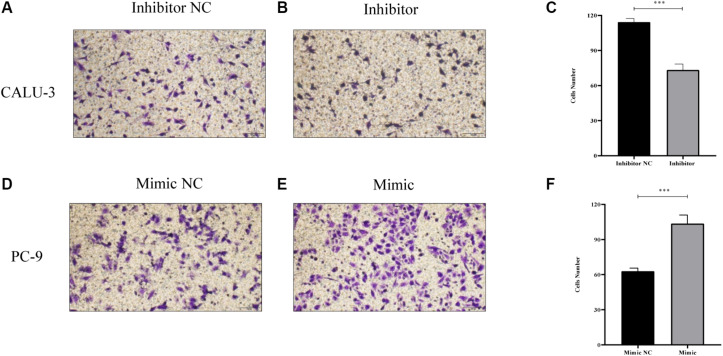
**(A–C)** Transwell migration assay without Matrigel was performed in miR-4687-3p inhibitor or inhibitor control transfected CALU-3 cells (Magnification ×400). **(D–F)** Transwell migration assay without Matrigel was performed in miR-4687-3p mimic or mimic control transfected PC-9 cells (Magnification ×400). ***p < 0.001.

MiR-4687-3p inhibitor and miR-4687-3p mimic was transfected into CALU-3 and PC-9 cells respectively, to down-regulate or up-regulate the miR-4687-3p level. The results showed that down-regulation of miR-4687-3p with inhibitor could markedly suppress the proliferation (Figure 7A), invasion (Figures 8A–C), and migration (Figures 9A–C) in CALU-3 cells compared to inhibitor NC, respectively. Overexpressed miR-4687-3p by using mimic could promote the proliferation (Figure 7B), invasion (Figures 8D–F), and migration (Figures 9D–F) of PC-9 cells, compared to the mimic NC, respectively.

## Discussion

In the study, serum miR-4687-3p was discovered up-regulated in NSCLC, compared to the healthy, and showed a remarkable differential diagnosis value for the first time. Zsófia Brigitta Nagy revealed that the plasma miR-4687-3p overexpressed in colorectal cancer when compared with tubulovillous adenoma, via microarray screening (Nagy et al., 2019). However, they did not conduct the further validation.

For diagnosis of NSCLC, compared with biopsy, the serum-based approach was found to have more advantages, such as easy to access and acceptable to patients. MiRNAs were demonstrated to play an important role in carcinogenesis and have the potential for diagnosis of multiple cancers (Cheng, 2015; Maia et al., 2015; Gao et al., 2016; Li et al., 2017; Romano and Kwong, 2018). Moreover, endogenous circulating miRNAs have attracted widespread attention (de Souza et al., 2017).

In the training cohort, microarray assay was used to screen the serum differentially expressed miRNAs (DEMs) between NSCLC patients and the healthy, directly. Furthermore, we sorted 60 serum samples to verify the differential expression and found that serum miR-4687-3p and serum miR-6087 owned a higher expression in the NSCLC group than that of the healthy. As to differential diagnostic ability, serum miR-4687-3p (AUC = 0.679) might be a potential diagnostic biomarker for NSCLC.

Abnormally expressed serum cancer-related miRNAs were usually closely related to its expression in cancer tissues. According to the TCGA NSCLC database, compared to the corresponding normal lung tissues, the miR-4687-3p was up-regulated in LUAD and LUSC tissues, which indicated that miR-4687-3p was NSCLC-related miRNA.

Otherwise, as a favorable NSCLC diagnostic biomarker, it is vital to certain biological function to participate in the occurrence and development of the NSCLC. In addition, serum miR-4687-3p has not been reported as a diagnostic biomarker in several malignancies. Research of miR-4687-3p participating in the occurrence and development of tumors has not been reported yet. Although the specific mechanism of the miRNA on mRNA was unascertained, it was explicated that miRNAs triggered target mRNA degradation by complementary combining to 3’ UTR of mRNA.

By bioinformatics analysis, we revealed that miR-4687-3p owned 3,851 target genes, which significantly enriched the TGF-β signaling pathway. Previous researches had reported that the TGF-β pathway regulated the invasion and migration of cancer cells via epithelial-mesenchymal transition (EMT) (Xu et al., 2009; Schneider et al., 2011; Ma et al., 2015; Zhao et al., 2018; Camerlingo et al., 2019) and TGF-β could form a complex with Smad4 and translocate into the nucleus to regulate gene transcription (Wang et al., 2015; Yang et al., 2019), which suggested that the TGF-β pathway was vital in carcinogenesis. Some specific molecules served as tumor promoter or suppressor and can regulate the cancer cells proliferation, invasion, and migration via the pathway (Colak and Ten Dijke, 2017; Seoane and Gomis, 2017). In the present study, the target genes enriched in the TGF-β pathway implied that the miR-4687-3p might serve as a promoter affected the carcinogenesis of NSCLC. Moreover, the results proved the above hypothesis. Down-regulation miR-4687-3p could suppress the proliferation, invasion, and migration of NSCLC cells, while overexpression miR-4687-3p had the opposite effects. Importantly, data from the present study revealed that miR-4687-3p as a tumor promoter could promote tumor growth.

In conclusion, our results strongly suggested that serum miR-4687-3p overexpressed in NSCLC and miR-4687-3p could promote the growth and migration of NSCLC cells, which demonstrated that serum miR-4687-3p could be a novel and favorable biomarker for NSCLC. Compared with those studies of circulating miRNAs in diagnosing NSCLC, our study is unique for the following reasons. First, we screened a large number of serum miRNAs via microarrays, which enabled us to identify potential diagnostic serum miRNAs. Furthermore, we compared the expression of tissue miR-4687-3p in NSCLC and the healthy based on the TCGA database. We found that miR-4687-3p possesses the potency to promote NSCLC cells growth, migration, and invasion. Our findings suggest that miR-4687-3p functions as a tumor promoter in NSCLC and holds promise as a prognostic biomarker and potential therapeutic target for NSCLC.

However, our study has some limitations. Currently, there is no standard endogenous control for circulating miRNA studies. The stable control (miR-484) needs to be validated in more studies. In this research, we evaluated the role of miR-4687-3p in NSCLC, but we did not identify the underlying molecular mechanisms. In future research, we will focus on the specific mechanism of miR-4687-3p.

## Data Availability Statement

We have submitted the microarray data to GEO repository (https://www.ncbi.nlm.nih.gov/geo/query/acc.cgi?acc=GSE15 7074).

## Ethics Statement

The studies involving human participants were reviewed and approved by the Zhengzhou University. The patients/participants provided their written informed consent to participate in this study. Written informed consent was obtained from the individual(s) for the publication of any potentially identifiable images or data included in this article.

## Author Contributions

ML conducted the research and writing the manuscript. LD and JL designed the study and contributed to the writing. QS assisting with the research. SO, ZZ, MW, and CZ provided the serum samples. TY, YW, XZ, and WX collected the serum samples and analysis the data. All authors read and approved the final manuscript.

## Conflict of Interest

The authors declare that the research was conducted in the absence of any commercial or financial relationships that could be construed as a potential conflict of interest.

## Footnotes

1 http://cancergenome.nih.gov

## Supplementary Material

Supplementary Table 1 The information for samples from training cohort.

Supplementary Table 2 The information for samples from validation cohort.

Supplementary Table 3 The information for differently expressed miRNAs between NSCLC and healthy based on the microarray (fold change > 1.5, *P* < 0.05).
