# Supplementary material for: Serum MiR-4687-3p Has Potential for Diagnosis and Carcinogenesis in Non-small Cell Lung Cancer
Source: Front Genet. 2020 Nov 23;11:597508. doi: 10.3389/fgene.2020.597508 (PMC7721467; doi:10.3389/fgene.2020.597508)
Supplement: Supplementary Table 1 — The information for samples from training cohort. [file Table_1.docx]

| **Sample NO.** | **Serum NO.** | **Sex** | **Age** | **Histopathological Type** |
| --- | --- | --- | --- | --- |
| **NSCLC** |  |  |  |  |
| 1 | 1-1 | Male | 48 | LUAD |
|  | 1-2 | Male | 48 | LUAD |
| 2 | 2-1 | Male | 52 | LUSC |
|  | 2-2 | Male | 55 | LUSC |
| 3 | 3-1 | Male | 57 | LUAD |
|  | 3-2 | Male | 59 | LUAD |
| 4 | 4-1 | Male | 61 | LUSC |
|  | 4-2 | Male | 61 | LUSC |
| 5 | 5-1 | Male | 65 | LUSC |
|  | 5-2 | Male | 65 | LUSC |
| 6 | 6-1 | Female | 47 | LUAD |
|  | 6-2 | Female | 48 | LUAD |
| 7 | 7-1 | Female | 50 | LUAD |
|  | 7-2 | Female | 52 | LUAD |
| 8 | 8-1 | Female | 55 | LUAD |
|  | 8-2 | Female | 56 | LUAD |
| 9 | 9-1 | Female | 58 | LUAD |
|  | 9-2 | Female | 61 | LUAD |
| 10 | 10-1 | Female | 61 | LUAD |
|  | 10-2 | Female | 62 | LUAD |
| **Healthy** |  |  |  |  |
| 1 | 1-1 | Male | 47 |  |
|  | 1-2 | Male | 48 |  |
| 2 | 2-1 | Male | 53 |  |
|  | 2-2 | Male | 53 |  |
| 3 | 3-1 | Male | 56 |  |
|  | 3-2 | Male | 56 |  |
| 4 | 4-1 | Male | 57 |  |
|  | 4-2 | Male | 58 |  |
| 5 | 5-1 | Male | 60 |  |
|  | 5-2 | Male | 61 |  |
| 6 | 6-1 | Female | 47 |  |
|  | 6-2 | Female | 49 |  |
| 75 | 7-1 | Female | 50 |  |
|  | 7-2 | Female | 51 |  |
| 8 | 8-1 | Female | 52 |  |
|  | 8-2 | Female | 53 |  |
| 9 | 9-1 | Female | 55 |  |
|  | 9-2 | Female | 55 |  |
| 10 | 10-1 | Female | 57 |  |
|  | 10-2 | Female | 60 |  |
| *Note: LUSC: Lung Squamous Carcinoma; LUAD: Lung Adenocarcinoma* | | | | |

SUPPLEMENT TABLE | 1 The Information for Samples from Training Cohort.

SUPPLEMENT TABLE | 2 The Information for Samples from Validation Cohort.

| **Sample No.** | **Sex** | **Age** | **Histopathological Type** |
| --- | --- | --- | --- |
| **NSCLC** |  |  |  |
| 1 | Female | 57 | LUSC |
| 2 | Male | 61 | LUSC |
| 3 | Male | 68 | LUSC |
| 4 | Female | 49 | LUAD |
| 5 | Female | 71 | LUAD |
| 6 | Male | 52 | LUSC |
| 7 | Male | 66 | LUSC |
| 8 | Male | 64 | LUAD |
| 9 | Male | 66 | LUAD |
| 10 | Female | 41 | LUAD |
| 11 | Male | 61 | LUSC |
| 12 | Male | 67 | LUAD |
| 13 | Female | 62 | LUAD |
| 14 | Female | 56 | LUAD |
| 15 | Male | 45 | LUAD |
| 16 | Female | 63 | LUAD |
| 17 | Male | 71 | LUSC |
| 18 | Male | 66 | LUSC |
| 19 | Female | 48 | LUAD |
| 20 | Male | 57 | LUAD |
| 21 | Male | 76 | LUSC |
| 22 | Male | 66 | LUSC |
| 23 | Male | 49 | LUSC |
| 24 | Male | 66 | LUSC |
| 25 | Male | 79 | LUSC |
| 26 | Female | 70 | LUAD |
| 27 | Male | 48 | LUAD |
| 28 | Male | 69 | LUAD |
| 29 | Female | 54 | LUAD |
| 30 | Female | 56 | LUAD |
| **Healthy** |  |  |  |
| 1 | Male | 59 |  |
| 2 | Male | 60 |  |
| 3 | Female | 57 |  |
| 4 | Female | 36 |  |
| 5 | Female | 44 |  |
| 6 | Male | 43 |  |
| 7 | Female | 67 |  |
| 8 | Male | 61 |  |
| 9 | Male | 56 |  |
| 10 | Female | 73 |  |
| 11 | Male | 80 |  |
| 12 | Female | 68 |  |
| 13 | Female | 39 |  |
| 14 | Female | 53 |  |
| 15 | Female | 42 |  |
| 16 | Male | 56 |  |
| 17 | Male | 44 |  |
| 18 | Male | 55 |  |
| 19 | Male | 31 |  |
| 20 | Female | 55 |  |
| 21 | Female | 33 |  |
| 22 | Male | 41 |  |
| 23 | Female | 60 |  |
| 24 | Male | 57 |  |
| 25 | Female | 75 |  |
| 26 | Female | 52 |  |
| 27 | Female | 55 |  |
| 28 | Male | 58 |  |
| 29 | Female | 56 |  |
| 30 | Male | 57 |  |
| *Note: LUSC: Lung Squamous Carcinoma; LUAD: Lung Adenocarcinoma* | | | |

SUPPLEMENT TABLE | 3 The Information for Differently Expressed MiRNAs Between NSCLC and Healthy Based on The Microarray (Fold Change > 1.5, *P* < 0.05).

| **Name** | ***P*-value** | **Fold Change** | | **Regulation** |
| --- | --- | --- | --- | --- |
| miR-1200 | 0.009153057 | 2.2658436 | up | |
| miR-1267 | 0.040648604 | 3.9578324 | up | |
| miR-1273h-5p | 0.00590599 | 3.003938 | up | |
| miR-1306-5p | 0.026837639 | 1.7541446 | up | |
| miR-16-2-3p | 0.001023155 | 2.353492 | up | |
| miR-1915-5p | 0.027462619 | 3.344667 | up | |
| miR-203b-3p | 0.008523768 | 1.962696 | up | |
| miR-2115-5p | 0.014051409 | 2.2533536 | up | |
| miR-214-5p | 0.046407578 | 2.1343655 | up | |
| miR-216a-3p | 0.030463844 | 2.2803436 | up | |
| miR-218-2-3p | 0.025151545 | 2.5359622 | up | |
| miR-219b-5p | 0.029786355 | 1.7063289 | up | |
| miR-3184-3p | 0.040066952 | 2.1534332 | up | |
| miR-3671 | 0.0373597 | 2.0384881 | up | |
| miR-3678-5p | 0.015730098 | 2.1607595 | up | |
| miR-412-5p | 0.038559064 | 2.2796096 | up | |
| miR-4258 | 0.013240185 | 2.4002711 | up | |
| miR-4277 | 0.002101804 | 2.829854 | up | |
| miR-4297 | 0.020892582 | 1.8231539 | up | |
| miR-432-3p | 0.042212586 | 2.3359796 | up | |
| miR-4488 | 0.021610562 | 2.4761787 | up | |
| miR-4500 | 0.033071997 | 1.8656669 | up | |
| miR-4503 | 0.044425628 | 2.7391626 | up | |
| miR-452-3p | 0.026783719 | 2.334152 | up | |
| miR-4528 | 0.038434042 | 2.2004917 | up | |
| miR-4529-5p | 0.009236916 | 2.4553845 | up | |
| miR-4638-3p | 0.037922986 | 2.9065989 | up | |
| miR-4646-3p | 0.036111062 | 1.7954945 | up | |
| miR-4667-3p | 0.007746138 | 4.2529389 | up | |
| miR-4687-3p | 0.010252594 | 1.5407708 | up | |
| miR-4716-5p | 0.014507646 | 4.4641027 | up | |
| miR-499a-3p | 0.00538572 | 1.8564304 | up | |
| miR-5010-3p | 0.007043346 | 3.8998158 | up | |
| miR-508-3p | 0.004538234 | 2.8842454 | up | |
| miR-520a-5p | 0.039517347 | 3.1340906 | up | |
| miR-548ad-3p | 0.022825615 | 2.3542129 | up | |
| miR-554 | 0.037631564 | 1.7416564 | up | |
| miR-5589-3p | 0.01214037 | 2.0706533 | up | |
| miR-5698 | 0.031779027 | 2.0172482 | up | |
| miR-5707 | 0.01712746 | 1.8436409 | up | |
| miR-590-5p | 0.016581389 | 2.2271868 | up | |
| miR-6087 | 0.001283567 | 1.5446137 | up | |
| miR-611 | 0.041368658 | 3.0836102 | up | |
| miR-625-3p | 0.010612952 | 4.8596381 | up | |
| miR-6503-5p | 0.00330121 | 2.5486282 | up | |
| miR-6792-3p | 0.024327182 | 3.3370248 | up | |
| miR-6804-3p | 0.00409109 | 2.7293548 | up | |
| miR-6841-3p | 0.015344057 | 2.8918855 | up | |
| miR-6884-3p | 0.019677293 | 3.3183283 | up | |
| miR-7160-3p | 0.005104311 | 2.0596497 | up | |
| miR-924 | 0.018956379 | 1.7535653 | up | |
| miR-939-3p | 0.03979085 | 2.3880676 | up | |
| miR-1261 | 0.012710279 | 1.5085527 | down | |
| miR-1284 | 0.025891205 | 2.3247007 | down | |
| miR-1287-5p | 0.002915706 | 3.6752867 | down | |
| miR-4510 | 0.025585408 | 2.6189927 | down | |
| miR-574-3p | 0.031635462 | 2.7266446 | down | |
